# Proadrenomedullin in Sepsis and Septic Shock: A Role in the Emergency Department

**DOI:** 10.3390/medicina57090920

**Published:** 2021-09-01

**Authors:** Andrea Piccioni, Angela Saviano, Sara Cicchinelli, Federico Valletta, Michele Cosimo Santoro, Tommaso de Cunzo, Christian Zanza, Yaroslava Longhitano, Gianluca Tullo, Pietro Tilli, Marcello Candelli, Marcello Covino, Francesco Franceschi

**Affiliations:** 1 Emergency Department, Fondazione Policlinico Universitario A. Gemelli, IRCCS, 00168 Roma, Italy; andrea.piccioni@policlinicogemelli.it (A.P.); sara.cicchinelli@policlinicogemelli.it (S.C.); fede.valletta@gmail.com (F.V.); michelecosimo.santoro@policlinicogemelli.it (M.C.S.); tomdecunzo@gmail.com (T.d.C.); christian.zanza@live.it (C.Z.); gianlucatullo@gmail.com (G.T.); pietro.tilli@policlinicogemelli.it (P.T.); marcello.candelli@policlinicogemelli.it (M.C.); marcello.covino@policlinicogemelli.it (M.C.); francesco.franceschi@policlinicogemelli.it (F.F.); 2 Dietetics and Clinical Nutrition Unit, Department of Internal Medicine, University of Genoa, IRCCS Polyclinic Hospital San Martino, 16132 Genoa, Italy; lon.yaro@gmail.com

**Keywords:** sepsis, septic shock, proadrenomedullin, MR-proADM, procalcitonin, emergency department

## Abstract

Sepsis and septic shock represent a leading cause of mortality in the Emergency Department (ED) and in the Intensive Care Unit (ICU). For these life-threating conditions, different diagnostic and prognostic biomarkers have been studied. Proadrenomedullin (MR-proADM) is a biomarker that can predict organ damage and the risk of imminent death in patients with septic shock, as shown by a large amount of data in the literature. The aim of our narrative review is to evaluate the role of MR-proADM in the context of Emergency Medicine and to summarize the current knowledge of MR-proADM as a serum indicator that is useful in the Emergency Department (ED) to determine an early diagnosis and to predict the long-term mortality of patients with sepsis and septic shock. We performed an electronic literature review to investigate the role of MR-proADM in sepsis and septic shock in the context of ED. We searched papers on PubMed^®^, Cochrane^®^, UptoDate^®^, and Web of Science^®^ that had been published in the last 10 years. Data extracted from this literature review are not conclusive, but they show that MR-proADM may be helpful as a prognostic biomarker to stratify the mortality risk in cases of sepsis and septic shock with different degrees of organ damage, guiding emergency physicians in the diagnosis and the succeeding therapeutic workup. Sepsis and septic shock are conditions of high complexity and have a high risk of mortality. In the ED, early diagnosis is crucial in order to provide an early treatment and to improve patient survival. Diagnosis and prognosis are often the result of a combination of several tests. In our opinion, testing for MR-proADM directly in the ED could contribute to improving the prognostic assessment of patients, facilitating the subsequent clinical management and intensive treatment by the emergency physicians, but more studies are needed to confirm these results.

## 1. Introduction

Sepsis and septic shock are life-threatening medical emergencies characterized by severe systemic inflammation and organ dysfunction due to an excessive response to infections that may lead to death [1,2,3,4,5,6]. The definition of sepsis includes a dysregulated systemic inflammation, acute multi-organ dysfunction (i.e., cardiovascular, respiratory, and renal systems), and a deregulated immune response to a microbial invasion of the blood that is responsible of organ failure [7,8,9,10]. The mortality rate ranges from 15–25% [7]. Septic shock is sepsis characterized by a state of hypotension and hyperlactatemia, refractory to adequate fluid volume resuscitation that leads to hypoperfusion abnormalities, oliguria, and the alteration of mental status [7]. Septic shock has a mortality rate that ranges from 30–50% [7]. The early identification of sepsis and septic shock is essential for immediate treatment [1,2] and for the reduction of the patient mortality rate [10,11]. Sepsis can affect people of all ages [2,3,4]. Therapy for sepsis should be personalized and tailored according to the patient’s needs. Many biomarkers such as procalcitonin (PCT) or interleukin (IL)-6 or IL-18 are used in clinical practice to facilitate the diagnosis of sepsis [5,6]. Novel biomarkers such as proadrenomedullin (MR-proADM), kallistatin, testican-1, and presepsin have been introduced to assess the severity of sepsis and to predict the organ damage and the risk of imminent death [5]. As of now, a golden standard biomarker in terms of the diagnosis and prognosis for sepsis and septic shock has not been found. The aim of our narrative review is to evaluate the role of MR-proADM as a biomarker of sepsis in the context of emergency medicine and to summarize the current knowledge about MR-proADM in sepsis and septic shock as a potential biomarker to achieve an early diagnosis and to predict the long-term mortality of patients directly in the ED.

## 2. Literature Research

We performed an electronic literature review to investigate the role of MR-proADM in sepsis and septic shock. We searched papers on PubMed^®^, Cochrane^®^, UptoDate^®^, and Web of Science^®^. No ethical approval was necessary to perform this review. The principal words we included in the search were “severe sepsis” OR “sepsis”, OR “septic shock”, AND “procalcitonin”, AND/OR “pro-adrenomedullin”, OR “MR-proADM”, AND/OR “IL-6”, AND/OR “systemic inflammation”, AND/OR “organ failure”, AND/OR “infections”, AND “diagnostic biomarkers” AND “prognostic biomarkers”, OR “bacteria-induced sepsis”, AND/OR Emergency Department (ED) OR Emergency Medicine. Our search was based on clinical trials, meta-analysis, randomized controlled trials, reviews, and systematic reviews if available. We extracted data from comprehensive studies based on the new definition of sepsis and septic shock and reviewed the role of serum MR-proADM as a diagnostic and/or prognostic biomarker according to the available literature. We summarized the main studies, exploring the role of MR-proADM with the investigated cut-off value (nmol/L) (when available). No exclusion criteria (patient age, gender, comorbidities, admission to ICU, etc.) were applied. We also searched studies performed in the context of emergency medicine/ED. Papers were initially selected by title and abstract and then by the availability of the full text. We reviewed the results of the studies on the basis of the total number of patients, levels of MD-proADM, outcomes, management in the ED or Intensive Care Unit (ICU), and use of MD-proADM or other biomarkers or stratification scores of the severity of the patient’s condition. We investigated a total of 16 manuscripts from 2013 to 2021. The limitation of our review is the heterogeneity of the studies (type of patients included, design of study, endpoints).

## 3. Role of MR-proADM in ICU and in ED

Several authors have investigated the role of MR-proADM in patients with sepsis and septic shock. MR-proADM is a stable and detectable fragment of 48-amino acids derived from ADM (a 52-amino acid peptide and member of the calcitonin family) that is mainly produced by vascular endothelial cells and smooth muscle cells. ADM and MR-proADM have effects on vasodilatation (on artery and vein), natriuresis, bronchodilatation, and they have influences on cardiac contractility and glomerular filtration [11], which are involved in some clinical manifestations of sepsis and septic shock as refractory hypotension. MR-proADM has a half-life that is longer than ADM and can be more easily detected in blood compared to ADM, which is rapidly cleared from the circulation.

Most of the reported studies found that MR-proADM was a reliable biomarker that could serve as an early predictor of high mortality risk. In fact, levels of MR-proADM can potentially reflect the severity of organ dysfunction, even in the first stages of the disease, in the progression of systemic inflammatory response, in the movement from sepsis to septic shock, and in the mortality risk of septic patients [11,12]. A prospective observational study conducted with 213 septic patients showed that MR-proADM was able to predict system dysfunction (respiratory, coagulation, renal, neurological, and cardiovascular) and was well-correlated with Sequential Organ Failure Assessment (SOFA) score components [13]. The same results were obtained by Onal et al. [11], who concluded that MR-proADM could be a good alternative to SOFA score. L. Buendgens and his team [14] designed a prospective study to assess the role of MR-proADM in a cohort of 203 ICU patients and 66 healthy controls that they followed for a period of 26 months. They demonstrated that MR-proADM values were higher in critically ill patients—especially in those with sepsis progression—with a close correlation with other markers of systemic inflammation and endothelial dysfunction. Moreover, MR-proADM levels correlated with scores for disease severity (Acute Physiology and Chronic Health Disease Classification System (APACHE II), SOFA, and Simplified Acute Physiology Score (SAPS2)). The best cut-off value that was found by these authors to identify patients at high mortality risk was of 1.4 nmol/L [14]. Similar results were also reported by Gonzales Del Castillo et al. [15] in a larger study of 684 patients admitted to the ED for a suspected infection.

The abovementioned authors found that MR-proADM was able to identify those hiding an underlying severe condition and who were at high risk for delayed or insufficient initial treatment. In addition, authors compared several biomarkers (MR-proADM, C-reactive protein (CRP), PCT, and lactate) and clinical scores (SOFA, quick SOFA, and National early warning score (NEWS)), concluding that MR-proADM could help identify patients with low NEWS or quick SOFA values but who were at high risk for sepsis progression, helping in the initial treatment choices [15]. A prospective observational study of 657 patients with an acute infection conducted by Haang et al. [16] reported that the combination of MR-proADM and SOFA-score would better improve the stratification risk of patients for 30-day mortality (area under the curve (AUC) 0.87) than the SOFA-score alone (AUC 0.81). The authors defined a MR-proADM threshold value of 1.75 nmol/L as a prognostic value for 30-day mortality (sensitivity 81%, specificity 75%, and negative predictive value 98%) [16].

Spoto et al. [2] conducted a study on 571 septic patients and reported that MR-proADM has a strong correlation with a high risk of 90-day mortality, with a cut-off of 3.39 nmol/L for septic patients and a cut-off value of 4.33 nmol/L for shock patients. In another prospective study of 209 patients with a clinical sepsis diagnosis, S. Spoto [17] et al. showed that MR-proADM had an important function in predicting the development of organ failure over 24 h [17]. Significant evidence of MR-proADM prognostic reliability was also provided by a prospective observational study conducted in a sample of 326 patients with sepsis or septic shock by Andaluz-Ojeda [18] and his coworkers. Their results showed that MR-proADM was an optimal biomarker for the early identification of patients who had a high-risk of mortality, even if these patients who initially had a moderate clinical severity [18]. Such evidence further strengthens the role of MR-proADM as a prognostic factor for mortality in critical illness, but this evidence also shows how its inclusion in the first evaluation of septic patients with a moderate clinical condition is able to predict later organ dysfunction. Schuetz et al. [19] conducted a prospective, multicenter study including 7132 patients and revealed that MR-proADM improved the models that predict ICU admission for patients with sepsis and septic shock. In a cohort of 128 septic patients in the ED, Travaglino et al. [20] proved that MR-proADM was correlated with the APACHE score. Chris-Crain et al. [21] proved that MR-proADM was a good prognostic biomarker in critically ill patients with sepsis. However, a neat cut-off value for the identification of septic patients with a high mortality risk has not yet been found, and more studies are needed to finally set a threshold that can be standardized.

## 4. Discussion

Sepsis and septic shock are medical emergencies that require a proper diagnosis and appropriate management from the moment of admission to the ED. In fact, sepsis and septic shock carry a high mortality risk for patients [7,10,16]. Many factors contribute to the complexity of these conditions. Among them, the over-activation and dysregulation of the innate immune system in response to a blood microbial invasion is a topic of great interest in order to better define the most targeted therapeutic strategy. The innate immune system expresses some receptors that are able to recognize the signaling of damage or infection as damage-associated molecular patterns (DAMPs) or pathogen-associated molecular patterns (PAMPs). DAMPs and PAMPs, which are binding receptors of the innate immune cells, lead to the release of many pro-inflammatory cytokines and molecules such as tumor necrosis factor-α (TNF-α), interleukin-6 (IL-6), and interleukin-1β (IL-1β) followed by the release of acute phase proteins such as CRP, PCT, and MR-proADM [22]. In the context of the ED, it is important to have easily measurable biomarkers that can produce an indication level of the patient’s severity level in order to modulate the priority and the intensity of the patient’s care. Literature studies [5,6] have investigated the role, both diagnostic and prognostic, of some biomarkers such as PCT, IL-6, IL-18, presepsin, etc., in patients with sepsis and septic shock without finding conclusive results. MR-proADM seems to be a good biomarker in assessing a patient’s initial state, evolution, and prognosis. Moreover, MR-proADM may be a good alternative to the sequential organ failure assessment (SOFA) score. In the context of the ED, especially in the case of overcrowding, the administration of a simple blood test may be more practical compared to the collection of a multitude of data to calculate a score. MD-proADM has shown a strong ability to predict localized bacterial infections and to make a differentiatial diagnosis of sepsis from SIRS in patients with hematologic malignancies [23]. More studies are underway to explore the pathophysiological profile of MD-proADM regarding the release kinetics and the blood clearance time in order to reduce the risk of false-positive or -negative results and to avoid confounding and misleading mistakes in interpretation.

Moreover, the best cut-off value of MR-proADM for the early diagnosis of sepsis and for predicting patient prognosis has not yet been clarified. More studies are required to better define it for use in clinical practice and directly in the ED.

Some other molecules proposed as ideal predictor biomarkers of sepsis include CD64, the soluble receptors of myeloid cells (sTREM)-1, the soluble urokinase-type plasminogen activator receptor (suPAR), and pentraxin-3, but they are still far from application in the ED. Recent advances in technology are now focusing on microbiome and non-coding RNAs [22], some of which, for example miR-21, contribute to inflammatory responses and multi-organ dysfunction (i.e., kidney, lung, and liver) during sepsis [24,25]. Moreover, several models and scoring systems involving the combination of biomarkers are in progress [26,27,28,29]. They seem to have interesting diagnostic and prognostic performance, but they need confirmation through more trials. Our review has some limitations. The analyzed studies often extrapolated conclusions about MR-proADM based on a combination of different biomarkers and/or scores and not from the analysis of MR-proADM alone. Moreover, most of research studies included in the present work were not conducted in the ED and were conducted in the ICU. The population sample and design of the studies were often not homogeneous. Due to these considerations, it is essential to perform more studies in the context of ED and to identify good biomarkers or a combination of biomarkers and their cut off values that are able to promptly recognize the different phenotypes of septic patients in order to stratify the most urgent patients in order to improve the quality of care and survival, starting directly from the ED. The summary of studies exploring the role of MR-proADM can be seen in Table 1.

## 5. Conclusions

Data extracted from this narrative literature review showed that MR-proADM may be helpful as a prognostic biomarker to stratify the mortality risk in cases of sepsis and septic shock with different degrees of organ damages directly in the ED. Sepsis and septic shock are conditions of high complexity and high mortality-risk. In the ED, early diagnosis is crucial to provide early treatment and to improve patient survival. Diagnosis and prognosis are often the result of a combination of several tests. In our opinion, testing MR-proADM directly in the ED could help emergency physicians to facilitate the subsequent clinical management and intensive treatment of septic patients with better patient survival results.

## Figures and Tables

**Table 1:** Summary of studies exploring the role of proadrenomedullin (MR-proADM).

Authors	Type of Study	Number of Patients and Time of Enrollment	Evidence	Cut-Off (nmol/L)
Spoto S [2] et al. Microb Pathog 2019	Retrospective observational study in adults	571 (2012–2018)	MR-proADM has a strong correlation with 90-day mortality	3.39 (for sepsis) and 4.33 (for septic shock)
Li [3] et al. Med Intensiva 2018	Systematic review and meta-analysis of thirteen studies in adults	2556 (1999–-2017)	MR-proADM might predict the prognosis of septic patients	unknown
Fahmey [4] et al. Korean J Pediatr 2018	Prospective observational pediatric study	60 septic newborns vs. 30 healthy neonates (May 2016–January 2017)	MR-proADM: valid biomarker for neonatal sepsis. High levels were associated with mortality and the disease’s outcome.	4.3
Enguix-Armada [12] et al. Clin Chem Lab Med 2016	Prospective observational study in adults	388 (2015)	MR-proADM is useful in the management of septic patients (measured in the first 24 h after ICU admission)	unknown
Andrés C [13] et al. Eur J Clin Invest 2020	Prospective observational study in adults	213 (2019–2020)	MR-proADM correlates with the largest number of Sequential Organ Failure Assessment (SOFA) score components and with organ dysfunction	1.4
Buendgens L [14] et al. Mediators Inflamm 2020	Prospective observational study in adults	269 (2018–2020)	MR-proAMD values are higher in critical septic patients and correlates with other markers of systemic inflammation and severity scores	0.05
Gonzalez Del Castillo J [15] et al. Crit Care 2019	Prospective observational study in adults	684 (May–July 2018)	MR-proADM identifies patients hiding an underlying severe condition and who are at high risk for delayed or insufficient initial treatment	1.77
Haag E [16] et al. Clin Chem Lab Med 2021	Prospective observational study in adults	657 (2019)	MR-proADM plus SOFA-score provide a better risk stratification than SOFA alone	1.75
Spoto S [17] et al. Sci Rep 2020	Prospective observational study in adults	209 (May 2014–June 2018)	MR-proADM anticipates organ failure in septic patients	1
Andaluz-Ojeda D [18] et al. Ann Intensive Care 2017	Prospective observational study in adults	326 (April 2013–January 2016)	MR-proADM predicts mortality in patients with sepsis at an early clinical stage	0.8
Schuetz [19] et al. Crit Care 2015	Review in adult patients	4 studies (March 2013–October 2014)	MR-proADM: prognostic marker that may improve site of care decisions	unknown
Kim [22] et al. Infect Chemother 2020	Review in adult patients	9 studies (1985–2020)	MR-proADM predicts 28-day mortality in septic patients	unknown
Al Shuaibi [23] et al. Clin Infect Dis 2013	Control observational study in adults	340 (June 2009–December 2010)	MR-proADM is useful in the management of febrile patients with hematologic malignancies. It localized bacterial infection and differentiated sepsis from SIRS	0.91 median level in septic patients (range: 0.05–8.78) 0.79 median level in non-septic patients (range: 0.05–6.48)
Valenzuela-Sánchez [25] et al. Minerva Anestesiol 2019	Prospective observational single-center study in adults	20 ICU-patients (June 2011–January 2013)	MR-proADM helped to identify sepsis in patients admitted to ICU. After 48 h of admission, it was associated with death risk	1.425 (before ICU admission) 5.626 (48 hours after)
Viaggi [26] et al. PLoS One 2018	Prospective observational study in adults	64 (12 March–25 June 2016)	MR-proADM anticipates the modification of several scores (SOFA, Pitt, and CPIS) related to organ dysfunction	1.1
De La Torre-Prados [27] et al. Minerva Anestesiol 2016	Prospective observational study in adults	100 (January–December 2011)	MR-proADM correlates with 28-day mortality in septic shock patients	unknown

## Data Availability

Not applicable.
